# High-temperature ab initio calculations on FeSi and NiSi at conditions relevant to small planetary cores

**DOI:** 10.1007/s00269-017-0875-4

**Published:** 2017-02-27

**Authors:** E. T. H. Wann, L. Vočadlo, I. G. Wood

**Affiliations:** Department of Earth Sciences, UCL, Gower Street, London, WC1E 6BT UK

**Keywords:** FeSi, Earth’s core, Mercury, Mars, *Ab initio*, NiSi, Terrestrial planets

## Abstract

The Fe–Ni–Si system is potentially a very important component of terrestrial planetary cores. However, at present, even the behaviour of the FeSi and NiSi end members is poorly understood, especially at low to moderate pressures—the data for FeSi are contradictory and NiSi has been little studied. For FeSi, there is general agreement that there is a phase transition from the ε-FeSi to the CsCl structure with increasing pressure, but, in experiments, there is disagreement as to the position and slope of the phase boundary and the range of coexistence of the two phases. In this paper we have used *ab initio* lattice dynamics calculations to determine the phase boundary between the ε-FeSi and CsCl structures as a function of pressure and temperature in both FeSi and NiSi. For FeSi, we find that the transition pressure at zero Kelvin is ~11 GPa and that the boundary between the ε-FeSi and CsCl phases varies little with temperature, having a slight negative Clapeyron slope, going from ~11 GPa at 300 K to ~3 GPa at 2000 K. For NiSi, there is much greater variation of the transition pressure with temperature, with a much shallower negative Clapeyron slope, going from ~156 GPa at 300 K to ~94 GPa at 2000 K.

## Introduction

It is widely accepted from cosmochemical and geophysical arguments that the Earth’s core, as well as the cores of the terrestrial planets, is comprised of mainly iron alloyed with nickel and a small proportion of light element(s). However, the identity of the light element(s) is still in question. Silicon has long been a popular candidate (Poirier 1994), and much work has been carried out to investigate the FeSi system and, to a lesser extent, the NiSi system (e.g. Lord et al. 2010; Vočadlo et al. 2012; Fischer et al. 2013). It has been suggested that pure FeSi may be found in the D′′ layer of the Earth, formed as a result of the reaction between the Fe-Ni liquid outer core and the MgSiO_3_ perovskite or post-perovskite of the lower mantle (Knittle and Jeanloz 1991). It has also been proposed that FeSi may form as a product of exsolution of the outer core during secular cooling (Buffett et al. 2000). Although it is more likely that a ternary Fe–Ni–Si alloy is to be found in the core of Earth and other terrestrial planets, investigating the ternary Fe–Ni–Si system is a significant undertaking. By first understanding the two binary end members, FeSi and NiSi, a good foundation is established for further investigation into the Fe–Ni–Si ternary.

Both experiments and calculations find only two stable structures in the FeSi system, the ε-FeSi phase and the CsCl phase (Vočadlo et al. 1999; Caracas and Wentzcovitch 2004; Lord et al. 2010; Fischer et al. 2013; Geballe and Jeanloz 2014). However, laser-heated diamond-anvil-cell (LH-DAC) experiments disagree as to the phase boundary of the transition (Lord et al. 2010; Fischer et al. 2013; Geballe and Jeanloz 2014). Both Fischer et al. (2013) and Geballe and Jeanloz (2014) find a vertical boundary between the two phases, although the two studies disagree on the transition pressure; Fischer et al. (2013) put the phase boundary at 42 GPa, while Geballe and Jeanloz (2014) find the transition occurring at ~30 GPa. Although both studies observe a two-phase stability field, Fischer et al. (2013) report a surprisingly large two-phase region between 14 and 42 GPa while Geballe and Jeanloz (2014) find a much smaller two-phase region, the size of which varies depending on the pressure medium used—in argon, the two-phase stability field exists between 23 and 30 GPa; in neon, between 30 and 32.3 GPa. In contrast to these two studies, Lord et al. (2010) find a negative Clausius–Clapeyron slope of −55 MPa/K, also from LH-DAC experiments. This slope is in agreement with multi-anvil press (MAP) experiments carried out by Dobson et al. (2002), who found the CsCl-FeSi phase to be stable from 24 GPa and 1950 ± 50 K. Computer simulations show a similar range of transition pressures: *ab initio* calculations carried out at 0 K in the Generalised Gradient Approximation (GGA) find the transition from ε-FeSi to CsCl-structured FeSi occurring at 13 GPa (Vočadlo et al. 1999), 40 GPa (Caracas and Wentzcovitch 2004) and 20 GPa (Zhang and Oganov 2010). Caracas and Wentzcovitch (2004) also carried out calculations using the Local Density Approximation (LDA) and found the transition to occur at 30 GPa.

In contrast to the FeSi system, recent *ab initio* calculations on the NiSi system, carried out at 0 K, found that a number of structures were stable, with different phases coming into stability as pressure increased. The sequence of stable NiSi structures originally proposed was as follows: MnP → *P*4/*nmm* (or the CuTi phase) → *Pbma*-I → *Pnma*-III (FeB) → CsCl, with transitions occurring at 23, 61, 168 and 247 GPa, respectively (Vočadlo et al. 2012). Following this work, Wood et al. (2013) found a new stable phase of NiSi in experiments carried out in a MAP. This new phase of NiSi, with *Pmmn* symmetry (an orthorhombic distortion of the tetragonal CuTi structure), had not originally been considered by Vočadlo et al. (2012), but further *ab initio* calculations revealed that this *Pmmn* phase was indeed more stable than either of the *P*4/*nmm, Pbma*-I or *Pnma*-III (FeB) phases, resulting in a much simpler phase diagram at 0 K. The new phase stability sequence in NiSi, therefore, became MnP → *Pmmn* → CsCl, with transitions occurring at 21 and 264 GPa (Wood et al. 2013). More recent static computer simulations by Gavryushkin et al. (2015) confirmed the stability of the Pmmn phase but also suggested that a tetragonally distorted (a/c ~ 0.8) CsCl-type structure formed above 213 GPa, becoming fully cubic above 522 GPa. In the static simulations of Vočadlo et al. (2012), it was, however, noticed that the enthalpy difference between the ε-FeSi structured form of NiSi and the thermodynamically stable phases was as small as 8–12 meV/atom for part of the pressure range considered, suggesting that the ε-FeSi structure might become stable at finite temperatures. A subsequent study using LH-DAC and synchrotron X-ray diffraction by Lord et al. (2012) confirmed that this was indeed the case, with a transformation to the ε-FeSi structure being observed at 12.5 GPa and 1550 K, prior to a transformation to the cubic CsCl structure at 46 GPa and 1900 K; no evidence for the large tetragonal distortion suggested by Gavryushkin et al. (2015) was found in the X-ray diffraction patterns of the quenched samples. More recently, a detailed experimental investigation of the NiSi phase diagram to ~65 GPa using both MAP and LHDAC techniques (Dobson et al. 2016) determined the boundaries between the MnP, Pmmn, ε-FeSi and CsCl phases. In particular, the ε-FeSi to CsCl boundary was found to have a Clapeyron slope of -67 MPa/K, with the ε-FeSi + CsCl + liquid invariant point occurring at ~33 GPa and ~2125 K. (this study also found that, when the MnP-structured material is compressed at 300 K, a transition to a further metastable phase of NiSi occurs, somewhere between 35 and 60 GPa, with the high-pressure structure corresponding to that labelled Pnma-II by Vočadlo et al. (2012) for which the transition was predicted to occur at ~42 GPa).

In the present paper, we report static calculations on the FeSi system to determine whether any of the structures found to be stable, or close to stable, in NiSi are also stable in FeSi. We then present lattice dynamics calculations to determine the phase boundary of the ε-FeSi → CsCl phase transition in both FeSi and NiSi at high temperatures and pressures, and compare these to the experimental results.

## Calculation method

The calculations presented here all make use of density functional theory, DFT (Hohenberg and Kohn 1964), within the generalised gradient approximation, GGA, using the VASP code (Kresse and Furthmuller 1996). All calculations were spin polarised with a PBE functional (Perdew et al. 1996); 14 electrons were treated as valence for Fe, 4 for Si and 10 for Ni. However, in all cases the magnetic moments went to zero, as has previously been found for FeSi (Moroni et al. 1999). Convergence tests were carried out for all calculations, to ensure an error of less than 0.001 eV per atom. For both FeSi and NiSi, k-point grids of 17 × 17 × 17 and 9 × 9 × 9 were used for the CsCl and ε-FeSi phases, respectively, with plane-wave cutoff energies of 600 eV for FeSi and 800 eV for NiSi. For the lattice dynamics calculations, electronic temperature was varied, being set to each temperature at which the phonons were calculated.

### Static calculations

For the FeSi system, static calculations, at effectively 0 K, were carried out on the known stable phases with the ε-FeSi and CsCl structures (with the convergence parameters given above). In addition, the structures of other phases investigated for NiSi (Vočadlo et al. 2012; Wood et al. 2013) were also used as starting points (see section “Stability of NiSi-structured phases in FeSi at 0 K”), with convergence criteria to ensure errors of less than 0.001 eV per atom. Geometry optimisation was performed, generating internal energy values for a set of specified volumes. Volumes for these calculations ranged from ~7 to 15 Å^3^ per atom, equivalent to pressures up to ~400 GPa. The resulting energy–volume values were then fitted to an integrated third-order Birch-Murnaghan equation of state to determine enthalpy as a function of pressure (see, e.g., Vočadlo et al. 1999).

### Lattice dynamics

Lattice dynamics calculations were carried out to determine the phase boundary of the ε-FeSi → CsCl phase transition in both FeSi and NiSi. At a given temperature, the vibrational free energy can be calculated from the phonon frequencies, which were obtained using the small-displacements or “frozen phonon” method as implemented in the program Phon (Alfè 2009). For any system, the most stable structure is that which has the lowest Gibbs free energy, G:

$$G=U+PV-TS=F+PV,$$

where *U* is the internal energy, *P* is the pressure, *V* is the volume, *T* is temperature, *S* is entropy and *F* is the Helmholtz free energy. The total Helmholtz free energy, *F*_total_, is a function of volume and temperature and can be split into two parts such that

$${{F}_{\text{total}}}\left( V,T \right)={{F}_{\text{perfect}}}\left( V,0 \right)~+{{F}_{\text{vib}}}\left( V,T \right),$$

where *F*_perfect_(*V*,0) is the total energy of the static system and *F*_vib_(*V*,T) is the vibrational free energy due to temperature (including the zero point energy). *F*_perfect_(*V*,0) is obtained through static calculations. *F*_vib_(*V, T*) can be written in terms of temperature and phonon frequencies

$${{F}_{\text{vib}}}\left( V,T \right)=~{{k}_{B}}T\underset{i}{\mathop \sum }\,\left[ \frac{\hbar {{\omega }_{i}}}{2{{k}_{B}}T}+\ln \left( 1-~{{e}^{-\frac{\hbar {{\omega }_{i}}}{2{{k}_{B}}T}}} \right) \right],$$

where *ω*_i_ is the phonon frequency, *k*_B_ is Boltzmann’s constant, *ħ* is Planck’s constant divided by 2*π*, and *T* is the temperature. In each case, Phon was used to generate a 2 × 2 × 2 supercell, and the necessary displacements to calculate the phonon frequencies and the resulting summation over the phonon modes were converged in vibrational Q-points (15 × 15 × 15 and 11 × 11 × 11 for the CsCl and ε-FeSi phases, respectively). In this way, the vibrational free energy can be calculated and hence the total Helmholtz free energy can be obtained (in the quasi-harmonic approximation, ignoring anharmonic contributions). Having specified a set of temperatures and volumes for the simulations, the pressure is then given by the derivative of the free energy with respect to volume at a constant temperature

$$P=~-{{\left( \frac{\text{d}F}{\text{d}V} \right)}_{T}}$$

and, therefore, fitting the isothermal *F*–*V* curve allows G to be determined. The *F*–*V* curves were fitted to integrated 3rd order Birch–Murnaghan equations of state and also, as a check on the fitting sensitivity, to sixth-order polynomials. Having determined the fitting parameters for *F*(V), *G* and *P* were then calculated at each temperature for a closely spaced set of appropriate volumes, and *G* was then plotted as a function of *P*. The transition pressures, defined by the intersection of the *G*(*P*) curves for each phase at each temperature, were then determined by inspection. The results from the two functions used to fit F(V) gave essentially identical results, leading to transition pressures which differed by <1 GPa for FeSi and <2 GPa for NiSi.

## Results and discussion

### Stability of NiSi-structured phases in FeSi at 0 K

**Fig. 1:**
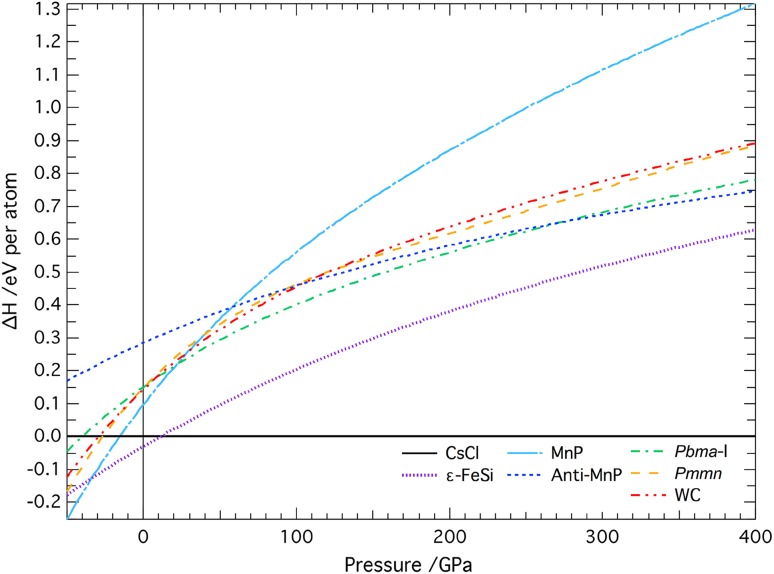
Plots of enthalpy, relative to that of CsCl-FeSi, against pressure for different FeSi structures, from static *ab initio* simulations using VASP. At *T* = 0 K, the enthalpy is equal to the Gibbs free energy, G and so the structure with the lowest enthalpy will be thermodynamically most stable. Transitions can be seen from the MnP structure to ε-FeSi at −34 GPa and from ε-FeSi to CsCl-FeSi at 11 GPa

Some of the stable NiSi structures seen in Vočadlo et al. (2012) were a result of spontaneous transformations after relaxation of the starting structure. In order to replicate these calculations as closely as possible, the same starting structures have been used here, with the exception of the NiAs, ‘anti’-NiAs and NaCl structures, which were all found to be unstable by a large margin in NiSi. Therefore, the structures that have been considered are the MnP, ‘anti-MnP’, Pbma-I and WC structures, as well as the new *Pmmn* phase found by Wood et al. (2013). Further details of these structures can be found in Vočadlo et al. (2012) and Wood et al. (2013). The calculated energy–volume values were fitted to third-order Birch-Muraghan equations of state to obtain the enthalpy–pressure values. A plot of the enthalpy differences (equivalent here to the differences in the Gibbs free energy), relative to that of CsCl-FeSi, shows that the only two phases that are stable in the FeSi system are the ε-FeSi and CsCl structures (see Fig. 1). The remaining structures all have a much greater enthalpy difference, of more than 0.1 eV per atom at all pressures, meaning that even at high temperatures, these phases are unlikely to become the most stable. The curve for the MnP-structure, however, shows a rapid decrease as pressure approaches zero; extrapolation to negative pressures shows that the MnP curve crosses the ε-FeSi curve at around −34 GPa (Fig. 1).

### Lattice dynamics calculations on FeSi

**Fig. 2:**
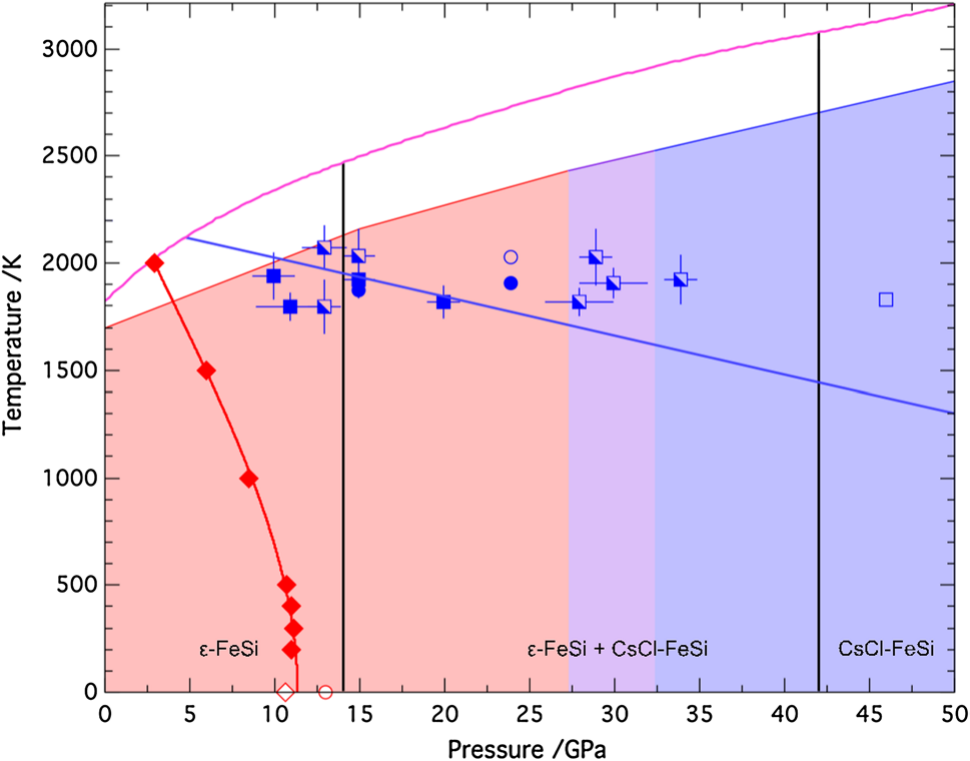
Phase diagram of FeSi, showing the calculated transition pressures from *ab initio* simulations using VASP (*red diamonds; filled* for lattice dynamics calculations and *open* for static calculations); the transition pressure determined by Vočadlo et al. (1999) is also shown (*red open circle*). As a guide to the eye, the phase *boundary line* has been described by an equation of the form *P* = *a* + *bT* ^2^ + *cT* ^3^, such that it has the correct asymptotic behaviour as required by thermodynamics (i.e. d*P*/d*T* tends to 0 as *T* tends to 0). Also plotted are the results from experimental studies. Blue squares are taken from Lord et al. (2010) and circles from Dobson et al. (2002); *filled symbols* indicate ε-FeSi structure only, *open symbols* indicate CsCl-FeSi and *half-filled* indicate a mixture of two phases. The phase boundary (*blue line*) as determined by Lord et al. (2010) has also been plotted. Also shown is the melting curve (*pink line*) of FeSi as measured by Lord et al. (2010). The phase diagram of Geballe and Jeanloz (2014) is shown as *shaded regions* to the limit of their experiments (*red area* for ε-FeSi, *purple area* for mixture of two phases and *blue area* for CsCl-FeSi). The phase boundaries of Fischer et al. (2013) are plotted as *straight black lines*

Lattice dynamics calculations on the ε-FeSi and CsCl phases were carried out for volumes of 8–12.5 Å^3^ per atom (equivalent to pressures up to ~400 GPa) and temperatures from 200 to 3000 K. However, only temperatures up to 2000 K were used in the subsequent analysis once it became clear that the calculated phase boundary crossed the melting line at higher temperatures. The transition pressures, listed in Table 1, were determined at each temperature from the intersection of the *G*–*P* curves, following the method detailed above (“Lattice dynamics”). The lattice dynamics calculations indicate that the phase boundary between the ε-FeSi and CsCl structures is very steep with a negative Clapeyron-slope. Figure 2 shows the calculated phase boundary, together with the results from three experimental studies. From Fig. 2, it can be seen that the pressure of the calculated phase boundary is lower than that in all experiments, but matches best with the lower bound of the two-phase region defined by Fischer et al. (2013) at 14 GPa. The Clapeyron slope of the calculated phase boundary matches best with the experimental slopes defined by both Fischer et al. (2013) and Geballe and Jeanloz (2014), who report a vertical phase boundary, in sharp contrast to Lord et al. (2010) who observed a much shallower Clapeyron slope.

**Table 1:** Calculated transition pressures from lattice dynamics for the ε-FeSi → CsCl transition in FeSi

Temperature (K)	Transition pressure (GPa)
200	11.0
300	11.1
400	11.0
500	10.7
1000	8.5
1500	6.0
2000	2.9

Estimates of the thermoelastic properties of both ε-FeSi and CsCl-structured FeSi can be also determined from the *F*(*V*) fits. The third-order Birch-Murnaghan equations of state parameters obtained at each temperature are shown in Table 2. It was found that for both structures, the incompressibility at zero pressure, *K*_0_, varies linearly with temperature with slope: d*K*_0_/d*T* = −0.0218(2) GPaK^−1^ and −0.03089(6) GPaK^−1^ for ε-FeSi and CsCl-structures, respectively. K′_0_ varies very little with temperature being ~4.2 for ε-FeSi and ~4.4 for CsCl-FeSi. The volumetric thermal expansion coefficient, at zero pressure, was obtained from the values of V_0_ shown in Table 2 using the method given in Vočadlo et al. (2002; Eqs. 1 and 3). Assuming a temperature-independent expansion coefficient, α_0_, this leads to values of α_0_ = 3.0(1) × 10^−5^ K^−1^ and 3.59(7) × 10^−5^ K^−1^ for the ε-FeSi and CsCl-structures, respectively. Experimental values for *K*_0_ at 300 K for ε-FeSi are generally in the range 172–176 GPa (Guyot et al. 1997; Ross 1996; Sarrao et al. 1994); for the CsCl phase of FeSi, *K*_0_ is not so well constrained with values of 184(5) GPa (Dobson et al. 2003), 225(2) GPa (Ono et al. 2007) and 223(9) GPa (Ono 2013) having been reported. In general, in FeSi, the values for *K*_0_ obtained from DFT simulations tend to be significantly greater than those obtained by experiment. For the ε-FeSi and CsCl structures respectively, values of *K*_0_ of 227 and 226 GPa (Vočadlo et al. 1999), 221 and 220 GPa (Caracas and Wentzcovitch 2004), 209 and 221 GPa (Moroni et al. 1999) have been reported. For ε-FeSi, the experimental values for the volumetric thermal expansion coefficient at zero pressure of 5.1(4) × 10^−5^ K^−1^ (Guyot et al. 1997) and 4.85(5) × 10^−5^ K^−1^ (Vočadlo et al. 2002) are ~60–70% greater than that obtained here from lattice dynamics. No corresponding experimental values for the thermal expansion coefficient of CsCl-FeSi are available at present. It would be of interest to determine the thermal expansion of CsCl-FeSi as the present calculations suggest that it might be greater than that for ε-FeSi, despite the CsCl phase being denser. The experimental value for d*K*_0_/d*T* in the ε-FeSi phase is −0.043(8) GPaK^−1^ (Guyot et al. 1997), approximately twice that obtained in the present work, although the difference is only marginally greater than might be expected from the uncertainties in the quantities. Once again, no corresponding values for CsCl-FeSi are apparently available at present.

**Table 2:** Birch–Murnaghan 3rd-order equation of state parameters from lattice dynamics for FeSi

T (K)	ε-FeSi structure			CsCl structure		
	*V*_0_ (Å^3^/atom)	*K*_0_ (GPa)	*K*_0_′	*V*_0_ (Å^3^/atom)	*K*_0_ (GPa)	*K*_0_′
200	11.083(5)	222.817(7)	4.24(1)	10.599(4)	232.768(7)	4.35(1)
300	11.105(5)	221.209(6)	4.22(1)	10.627(5)	229.789(7)	4.36(1)
400	11.133(5)	218.588(7)	4.23(1)	10.658(5)	226.746(7)	4.37(1)
500	11.161(6)	216.210(7)	4.24(1)	10.692(5)	222.544(8)	4.38(2)
1000	11.314(7)	205.496(8)	4.25(2)	10.876(7)	208.190(9)	4.41(2)
1500	11.478(10)	195.004(10)	4.27(2)	11.081(10)	192.588(11)	4.45(2)
2000	11.662(14)	183.590(12)	4.29(2)	11.306(14)	177.294(14)	4.49(3)

### Lattice dynamics calculations in the NiSi system

**Fig. 3:**
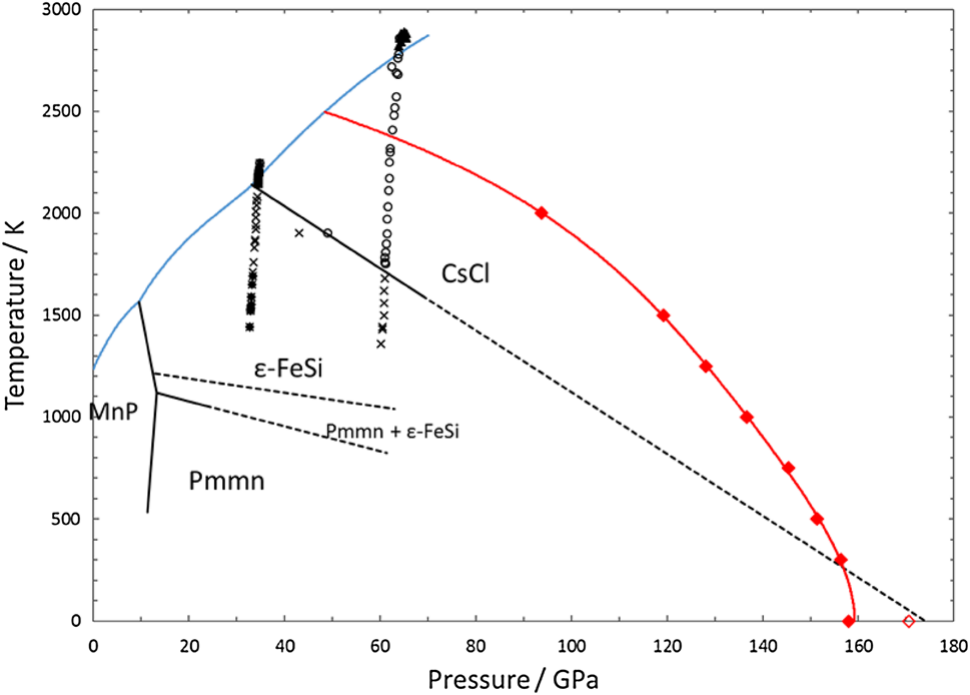
The phase diagram of NiSi, showing (in *red*) the calculated phase boundary between the ε-FeSi and CsCl structures [*filled red diamonds* from this study, including also a static calculation; *open red diamond* from Vočadlo et al. (2012)]. As a guide to the eye, the phase *boundary line* has been described by an equation of the form *P* = *a* + *bT* ^2^ + *cT* ^3^ + *dT* ^4^ such that it has the correct asymptotic behaviour as required by thermodynamics (i.e. d*P*/d*T* tends to 0 as *T* tends to 0). The experimentally determined melting curve is shown in *blue* (Lord et al. 2014) and the boundaries of the MnP, *Pmmn*, ε-FeSi and CsCl phases in *black* (from Dobson et al. 2016, with symbols defined therein). The ε-FeSi to CsCl phase boundary from these experiments is dashed to show extrapolation to zero K

As with the FeSi system, the phase boundary between the ε-FeSi and CsCl-structured phases of NiSi was calculated using the lattice dynamics method. Volumes between 6 and 11 Å^3^ per atom were used, equivalent to pressures of up to 400 GPa, and temperatures ranged from 300 to 3000 K. As with the FeSi calculations, only temperatures up to 2000 K were used in the analysis due to the phase boundary approaching the melting line at higher temperatures. The calculated transition pressures, determined in the same way as for FeSi, are listed in Table 3 and shown in Fig. 3. Figure 3 indicates that the calculated phase boundary corresponds reasonably well with the experimentally constrained phase boundary (Lord et al. 2012, 2014; Dobson et al. 2016) in that both calculations and experiments show a negative Clapeyron slope of similar magnitude at high temperature, and a similar transition pressure at low temperature. The experimental data are, however, fairly sparse. Additionally, there is good agreement in the transition pressure at 0 K between the calculations carried out here, the static computer simulations of Vočadlo et al. (2012) and a linear extrapolation of the experimental phase boundary (Dobson et al. 2016).

**Table 3:** Calculated transition pressures from lattice dynamics for the ε-FeSi → CsCl phase transition in NiSi

Temperature (K)	Transition pressure (GPa)
0	158.0
300	156.3
500	151.4
750	145.4
1000	136.6
1250	128.1
1500	119.2
2000	93.8

As for FeSi, estimates of the thermoelastic properties of both ε-FeSi and CsCl-structured NiSi were determined from the *F*(*V*) fits. The third-order Birch–Murnaghan equation-of-state parameters obtained at each temperature are shown in Table 4. For both ε-FeSi and CsCl structures, the incompressibility at zero pressure, K_0_, varies linearly with temperature giving *d*K_0_/*d*T = −0.0333(1) and −0.0389(4) GPaK^− 1^ for ε-FeSi and CsCl-structured NiSi, respectively. Again, K_0_′ varies little with T, being ~4.5 and ~4.7 for ε-FeSi and CsCl-structured NiSi, respectively. The volumetric thermal expansion coefficient at zero pressure gave values of *α*_0_ = 5.0(1) × 10^−5^ K^−1^ and 7.0(2) × 10^−5^ K^−1^ for the ε-FeSi and CsCl-structured NiSi, respectively. Corresponding experimental values for NiSi are extremely limited as both of these structures are only found at high pressures. Lord et al. (2012) determined the equation of state of both phases at 300 K reporting a value for the ε-FeSi structure of *K*_0_ = 161(3) GPa and *K*_0_′ = 5.6 for a fixed *V*_0_. Although the calculated value presented here [181.10(1) GPa] is higher than the experimental value, it is in excellent agreement with the result from the athermal simulations [181.143(4) GPa] of Vočadlo et al. (2012). For CsCl-structured NiSi it is difficult to make a meaningful comparison with experiments, since these give values ranging from ~150 < *K*_0_ < 240 GPa depending on the details of the data analysis (Lord et al. 2012). There are no experimental data with which to compare our estimates of either the volumetric thermal expansion coefficient at zero pressure or dK_0_/dT, although we note that, as for FeSi, the CsCl-structured material has the higher thermal expansion coefficient.

**Table 4:** Birch–Murnaghan 3rd -order equation of state parameters from lattice dynamics for NiSi

T (K)	ε-FeSi structure			CsCl-FeSi structure		
	*V*_0 _(Å^3^/atom)	*K*_0_ (GPa)	*K*_0_′	*V*_0 _(Å^3^/atom)	*K*_0_ (GPa)	*K*_0_′
300	11.69(1)	181.10(1)	4.36(2)	11.732(9)	158.028(6)	4.59(1)
500	11.79(2)	174.56(1)	4.39(2)	11.87(1)	149.894(7)	4.63(1)
750	11.92(2)	166.51(1)	4.41(2)	12.05(2)	139.937(9)	4.67(2)
1000	12.06(2)	158.00(1)	4.45(2)	12.25(2)	130.03(1)	4.72(2)
1250	12.21(3)	149.71(2)	4.47(3)	12.46(3)	120.52(1)	4.76(3)
1500	12.38(3)	141.23(2)	4.51(3)	12.69(4)	111.12(2)	4.80(3)
2000	12.74(5)	124.54(2)	4.58(4)	13.21(7)	92.76(2)	4.90(5)

## Discussion and conclusions

Calculations on the NiSi-structured phases in FeSi confirm that there are only two stable phases, those with the ε-FeSi and CsCl structures. The large energy gap separating the other structures means that, although these are static calculations (0 K), it is unlikely that any of these will become the most stable phase at high temperatures. However, extrapolation of the H–P curves to negative pressures shows that the MnP-phase becomes stable at −34 GPa. Taking this into account, the stability sequence in FeSi with increasing pressure then becomes MnP-FeSi → ε-FeSi → CsCl-FeSi. This stability sequence is also observed in RuSi, OsSi and CoSi, as predicted by *ab initio* static calculations similar to those carried out here (Hernandez et al. 2015). As with FeSi, Hernandez et al. (2015) find the MnP structure in these compounds is only stable at negative pressures at 0 K, with transition pressures of −15, −6.3 and −10 GPa respectively for RuSi, OsSi and CoSi, indicating that (at 0 K) the MnP phase is stabilised as one descends Group 8 from Fe to Os. A similar pattern is seen with the Group 9 elements. The calculations of Hernandez et al. (2015) did not extend past 300 GPa, and so the CsCl-structured phase was not found in either RhSi or IrSi, but it is possible that the same MnP → ε-FeSi → CsCl-FeSi stability sequence would be seen at higher pressures. However, for the silicides of the group 10 elements, Hernandez et al. (2015) found a different, possibly more complicated, structural sequence as exemplified by experiments and calculations on NiSi (Vočadlo et al. 2012; Lord et al. 2012; Wood et al. 2013; Dobson et al. 2016).

Lattice dynamics calculations show that the calculated phase boundary between the ε-FeSi and CsCl phases in FeSi is lower in pressure at all temperatures than the experimental phase boundaries. The calculated phase boundary is similar to the lower bound proposed by Fischer et al. (2013), being near vertical with a negative Clapeyron slope, but in sharp contrast to that of Lord et al. (2010) who find a boundary with a much shallower negative Clapeyron slope. Indeed, it should be noted that none of the published DFT simulations support a shallow negative Clapeyron slope, as even the highest predicted transition pressure (that of Caracas and Wentzcovitch 2004: 40 GPa at zero K) is much lower than would be expected on the basis of extrapolation of Lord et al.’s experimental phase boundary. That we find lower transition pressures in the computer simulations might suggest that there is an element of kinetic inhibition in the experiments, which may also explain the differences in the experimental phase boundaries observed. Evidence of kinetic inhibition can be seen in the experiments themselves—each of the experimental studies reports some degree of metastable persistence of ε-FeSi, with Lord et al. (2010) finding only one instance, at 46 GPa and 1830 K, where the transition reaches completion to yield pure CsCl-FeSi. Differences in the size of the two-phase stability field observed in experiments may then be due to experimental differences that affect whether complete transformation could take place.

However, kinetic inhibition alone may not be sufficient to explain the large difference seen between experiments. Another credible explanation is the possibility of Si diffusion during heating. Dobson et al. (2002) used electron microprobe analysis to determine the exact stoichiometry of their samples, but this was not done in the LH-DAC experiments. It is possible that the stoichiometry of the samples differed sufficiently in the LH-DAC experiments which, when combined with the effect of kinetic inhibition, is enough to explain the large discrepancies between them. Giving credence to this argument is the fact that experiments have shown that small changes in composition affect the structure adopted by RuSi, an analogue material of FeSi; a small excess of Ru (~1%) favours the CsCl-structure, whereas samples deficient in Ru show a mixture of ε-FeSi and Ru_2_Si_3_ structures (Buschinger et al. 1997; Vočadlo et al. 2000).

In contrast to FeSi, although the experimental data are sparse, both the calculated and experimentally determined phase boundaries in NiSi show moderate negative Clapeyron slopes. The experimental (Dobson et al. 2016) and calculated transition pressures agree well at high pressures and low temperatures, but less so at lower pressures and high temperatures where anharmonic effects may become important and methods based on lattice dynamics may begin to fail. The fact that FeSi and NiSi behave so differently makes it difficult to predict which phases would be stable in the Fe–Ni–Si system—a composition more likely to be found in the cores of terrestrial planets than either pure FeSi or pure NiSi. Although at the extreme conditions of the Earth’s core—temperatures in the region of 6000 K and pressures up to 360 GPa (Anzellini et al. 2013)—the CsCl-structured phase is stable for both FeSi and NiSi, at lower pressures (e.g., <40 GPa) the two systems have very different phase diagrams, with the ε-FeSi and CsCl structures being stable in FeSi, and the MnP, *Pmmn*, ε-FeSi and possibly even the CsCl structures being stable in NiSi. This becomes particularly relevant when considering the cores of smaller terrestrial planets such as Mercury and Mars, where core pressures are much lower than in the Earth, with the core of Mars estimated to be between 24 and 42 GPa and 2000 and 2600 K (Fei and Bertka 2005), while the core of Mercury is thought to be between 8 and 40 GPa, and 1700–2200 K (Chen et al. 2008). It is, therefore, important both to understand exactly how and why phase stability differs in FeSi and NiSi and to properly investigate the Fe-Ni-Si ternary phase diagram.
